# Alpine dwarf shrubs on green roofs: species-specific physiological responses to substrate depth and drought intensity

**DOI:** 10.1007/s11104-026-08836-6

**Published:** 2026-07-09

**Authors:** Carlotta Musso, Friederike Barkmann, Johannes Rüdisser, Stefan Mayr, Andrea Ganthaler

**Affiliations:** 1 Department of Botany, University of Innsbruck, Sternwartestraße 15, 6020 Innsbruck, Austria; 2 Department of Ecology, University of Innsbruck, Sternwartestraße 15, 6020 Innsbruck, Austria

**Keywords:** Drought, Heat stress, Ericaceae, Anisohydric, Isohydric, Leaf water potential

## Abstract

**Background and aims:**

Green roofs can enhance urban biodiversity when local, functionally diverse species are used. However, extreme roof conditions require species selection based on plant eco-physiological characteristics.

**Methods:**

This study evaluated the performance of four Alpine dwarf shrubs (*Arctostaphylos uva-ursi*, *Vaccinium myrtillus*, *V. vitis-idaea*, *Calluna vulgaris*) in green roof plots with 10 and 30 cm substrate under natural conditions (temperate climate) and compared it with the drought-tolerant shrub *Salvia officinalis* as a reference. Seasonal and daily variations in leaf temperature, water potential, and gas exchange were recorded during humid and dry periods to assess how substrate depth modulates plant-soil interactions.

**Results:**

More favourable water availability and temperature with 30 cm substrate reduced drought stress and increased stomatal conductance, photosynthesis, and biomass in all shrubs compared to 10 cm substrate. However, species responded differently to substrate depth and drought intensity, with *A. uva-ursi* and *V. vitis-idaea* performing best: the former remained above its hydraulic vulnerability threshold (Ψ_50_) in both plots, and the latter with 30 cm substrate. Despite strict stomatal control, *C. vulgaris* and *V. myrtillus* exceeded their Ψ_50_ during drought in the 10 cm plot and experienced dieback. *S. officinalis* surpassed all Alpine species by achieving the highest gas exchange, growth and favourable water status in both substrates.

**Conclusions:**

Alpine dwarf shrubs can broaden the species for temperate green roofs, but some may experience drought-induced dieback with shallow substrate. Alternating deep and shallow substrate patches may be a cost-efficient strategy to improve shrub survival and account for species-specific limitations.

**Supplementary Information:**

The online version contains supplementary material available at https://doi.org/10.1007/s11104-026-08836-6.

## Introduction

In recent years, the installation of green roofs has increased worldwide (Ürge-Vorsatz et al. 2025; Saqib et al. 2024; Shahmohammad et al. 2022) with the aim of mitigating the negative effects of soil sealing (Butler et al. 2012). The increased use of green roofs is fostered by the documented environmental, economic and social benefits that this technology (*i.e.*, in this sense, green roofs are considered a green or sustainable technology) brings to urban areas. In fact, they have been reported to reduce the energy costs for heating or cooling buildings (Aleksejeva et al. 2024; Köhler et al. 2002), reduce noise pollution (Mihalakakou et al. 2023; Cook & Larsen 2021), retain pollutants leading to improved storm-water quality (Aleksejeva et al. 2024; Nowak et al. 2014; Köhler et al. 2002), and improve urban management of water runoff (Du et al. 2019; Berndtsson 2010). At the same time, they can increase the amount of green space in urban areas and enhance their aesthetic value (Thorpert et al. 2024).

Two main types of green roofs can be distinguished: extensive, and intensive (Shahmohammad et al. 2022; Cascone 2019; Besir and Cuce 2018). Extensive green roofs require low maintenance and are at low costs. They are characterised by shallow substrates between 5 and 15 cm and are mainly planted with annual and perennial herbaceous plants and grasses (Baraldi et al. 2019). Extensive roofs are the most common, as their weight is lower than that of intensive roofs (Técher et al. 2024; Oberndorfer et al. 2007). Intensive roofs have a deeper substrate and can also be planted with large shrubs and trees (Mihalakakou et al. 2023), thus they weigh and cost more.

Green roofs usually consist of a lightweight mineral substrate, a drainage and moisture retention layer, and a root-resistant waterproofing barrier (Orsi et al. 2025). They are designed to facilitate the establishment of the plants growing in the substrate layer (Raimondo et al. 2015; Emilsson and Rolf 2005). However, vegetation on green roofs, especially extensive green roofs, face extreme environmental conditions, such as high temperatures and irradiance, high winds, and fluctuations in water availability (Ganthaler et al. 2025; Frisk and Hanslin 2025; Baryła et al. 2019; Farrell et al. 2013). As a result, only certain species are suitable for planting on green roofs (Meetam et al. 2020), and plants with low, mat-forming, or compact growth, and with stress-tolerant characteristics are preferred for green roof planting due to their higher chance of survival (Oberndorfer et al. 2007). The most frequently used plant species on extensive green roofs are *Sedum* species (Liao et al. 2025), succulents with respective water storage capacity and CAM-photosynthesis under drought conditions (Nektarios et al. 2015). However, a wider use of local species could increase the functional diversity of the vegetation on the roof and promote plant establishment, as such species are adapted to the local climate (Ksiazek-Mikenas et al. 2021). Also, local plants are often aesthetically pleasing and form a continuum with the surrounding landscape (Butler et al. 2012; Monterusso et al. 2005). The inclusion of different life forms, such as woody shrubs, can further increase structural diversity and species richness (*e.g.*, by supporting animal communities; Cook-Patton 2015).

Dwarf shrubs are an important component of the Alpine vegetation at high elevations (Körner 2021). Due to their compact and low growth, dwarf shrubs cause more favourable conditions near the surface, creating their own microclimate and benefiting from the protection of snow cover (Ganthaler et al. 2022; Wilson et al. 1987). The environmental conditions experienced by plants at high elevations, such as extreme temperatures, high radiation, drought stress, and rapid changes in soil moisture, are similar to those on a green roof. Thus, dwarf shrubs, which naturally thrive under such harsh conditions, and in shallow, rocky soils with low water-holding capacity (Körner 2023), may be suitable species on green roofs. However, to date, no study has tested Alpine dwarf shrubs as potential candidates for green roof plantings and, in general, shrubs on green roofs are still understudied compared to succulents and herbaceous species (Savi et al. 2016a, b). The selection of appropriate plant species for green roofs can benefit from an eco-physiological approach (Savi et al. 2016a, b), including analyses of plant carbon and water relations. To understand a plant’s reaction to drought stomata regulation is important and its balance of water relations and photosynthesis as well as growth (Pirasteh-Anosheh et al. 2016). Plants growing on green roofs must be able to survive droughts but should also use more water during rainfall to increase rainwater retention (Chu and Farrell 2021). In this respect, isohydric species, characterised by a more conservative water strategy with tight stomatal control during dry periods, may be good candidates to withstand droughts on the green roofs. Conversely, anisohydric species which can increase the roof’s overall evapotranspiration, may be better suited to short-term droughts (Chu and Farrell 2021; Raimondo et al. 2015).

In the present study, we analysed whether four Alpine dwarf shrub species (*Arctostaphylos uva-ursi*, *Vaccinium myrtillus*, *V. vitis-idaea* and *Calluna vulgaris*) are suitable for green roofs and whether they are physiologically adapted to the limited water availability on green roofs. Particular focus was placed on how substrate depth influenced soil water availability and, thus, plant-soil interactions. In an experimental setup, the shrubs were planted at two different substrate depths (10 and 30 cm), chosen as representative to reflect the conditions of an extensive and an intensive green roof, respectively. For comparison, the Mediterranean shrub *Salvia officinalis* was also included, which is often used on Mediterranean green roofs, and known for its drought-tolerant behaviour (Raimondo et al. 2015). This species served as a reference for drought-tolerance against which the physiological responses of the Alpine dwarf shrubs could be compared. We monitored climatic conditions and analysed seasonal variations in leaf water potential, leaf temperature, transpiration, and photosynthesis (including both humid and dry periods), followed by damage assessments and plant biomass analyses.

We hypothesised that (i) Alpine dwarf shrub species can grow on green roofs but growth and vitality is species-specific; (ii) Alpine dwarf shrubs maintain low stomatal conductance and photosynthetic rate during drought; (iii) the drought resistance of dwarf shrubs and their physiological responses to drought (stomatal conductance, photosynthetic rate), are reflected in their growth rate on the roof. As such, low growth may result from limited stomatal conductance and carbon gain under periods of drought; (iv) the performance (in terms of vitality and growth) of dwarf shrubs is higher on deeper substrate, which is especially relevant under drought.

## Materials and methods

### Plant material and study design

In October 2023, 20 individuals of each species (the evergreen Alpine dwarf shrubs *Arctostaphylos uva-ursi* (L.) Spreng*.*, *V. vitis-idaea* L., and *Calluna vulgaris* (L.) Hull and the deciduous *Vaccinium myrtillus* L., together with the evergreen Mediterranean shrub *Salvia officinalis* L., for comparison; Suppl. Table S1) were planted randomly in two experimental green roof plots with different substrate depths (10 and 30 cm; 10 plants per species and plot) situated in the Botanical Garden of the University of Innsbruck (Tyrol, Austria, 620 m above sea level, a.s.l., 47°27’ N, 11°38’ E). All plants were purchased as 2–3-year-old juvenile plants in 10 cm pots from Bruns Pflanzen-Export GmbH & Co.KG, Bad Zwischenahn, Germany and had a size of about 7 cm in height (*A. uva ursi* with prostrate growth) and 10–15 cm (all other species). The plots were 2.5 m long and 1.5 m wide. This setup created contrasting conditions to examine the influence of substrate depth on soil water availability, as well as the effect of species-specific water use on soil water potential over the season. The use of two larger plots, rather than multiple smaller units, allowed for sufficient spacing between shrubs and reduced the variability of conditions towards the plot edges. Both plots were constructed like a green roof with several layers, including a drainage layer with an undulating design to facilitate water retention, below filter fleece, and the top substrate layer. The substrate comprises mineral and organic fractions. The mineral component, mainly brick chippings, contributes to structural stability and drainage. The organic fraction consists of green waste compost, supplying nutrients and promoting microbial activity (Weiss+Appetito 2025). Physical properties of the substrate include a pH of 7.5, dry bulk density of 950 kg/m^3^, water retention capacity of 45% of substrate volume, air content at full saturation of 10% of substrate volume, and salt concentration of 1.5 g/L. The water retention curves for the present substrate were recently measured by Ganthaler et al. (2025), reporting a saturated water content of 50, the permanent wilting point reached at a water content of 8, and the field capacity at 34 vol%.

In 2024, the mean annual temperature in Innsbruck was 11.4°C, and the mean annual precipitation was about 960 mm (GeoSphere Austria 2025). During the experiment, which took place in the vegetation period of 2024, the shrubs only received water by rain and were not additionally irrigated. For 18 days in August 2024 (from 13 to 30 August 2024), a rain exclusion roof with UV transparent greenhouse foil was installed to simulate an intense drought period. The installation of the shelter was necessary as, despite a natural drought period at this time, local evening thunderstorms and respective local, short-term precipitation were possible, which would have biased analyses of plant drought responses.

### Measurements

#### Climatic and soil conditions

On both plots, (micro-)climatic parameters were recorded. To monitor solar radiation (PAR; µmol m^−2^ s^−1^), a data logger system (Minikin RTHi/QTHi, EMS Brno, Czech Republic) was installed next to the plots with a measurement interval of 15 min. In addition, data on precipitation, air temperature (T_air_), and vapour pressure deficit (VPD; kPa) were available from a climate station located less than 1 km away. Soil conditions, including soil water potential (Ψ_soil_; MPa) and temperature (T_soil_; °C), were measured every 30 min using Teros 21 sensors (Meter group, Washington, USA), which were attached to data loggers (MicroLog SDI-12, EMS Brno, Czech Republic).

#### Leaf water potential and leaf temperature

Seasonal variations in midday leaf water potential (Ψ_L midday_; MPa) and midday leaf temperature (T_L midday_; °C) were monitored during the study. Additional detailed hydraulic (and gas exchange, *see below*) measurements, including predawn leaf water potential (Ψ_L pd_; MPa) and Ψ_L midday_, were taken during a humid period (27 May to 30 July 2024) and during a very dry (31 July to 29 August 2024) period. Ψ_L pd_ and Ψ_L midday_, were measured on four end twigs or leaves (in the case of *S. officinalis*) per shrub species, time point, and plot using a Scholander pressure chamber (mod. 1505D, PMS Instruments Company, Oregon, USA). Cut samples were covered with cling film and placed in sealed plastic bags to avoid desiccation until measurements (maximum time between harvest and measurement was 30 min). T_L midday_ of 4 to 6 individuals per shrub species, per plot and per time point, and the surface temperature of the substrate of the two plots, were recorded at midday using an infrared thermometer (IR 650-12D, Voltcraft, Bavaria, Germany).

#### Leaf gas exchange, quantum yield and electron transport rate of photosystem II

Seasonal variations in the midday values of net photosynthetic assimilation rate (A_midday_; µmol m^−2^ s^−1^) and in the stomatal conductance to water vapour (g_s midday_; mmol m^−2^s^−1^) were monitored. Additional detailed measurements were performed in the morning (from 08.00 to 10.00 h) and at midday (from 11.30 to 13.30 h) during the humid and dry periods, using a Li-6800 Portable Photosynthesis System (LI-COR Biosciences, Lincoln, NE, USA). During the measurements, the CO_2_ concentration was set to 420 ppm, the relative humidity to 55%, and the air flow was 500 mmol s^−1^. The fan speed was 10,000 rpm, and the light intensity was 1500 µmol m^−2^ s^−1^ but regulated according to the ambient light intensity (900–1000 µmol m^−2^s^−1^ in the morning). In addition, the quantum yield (Φ_PSII_) and electron transport rate (ETR; µmol m^−2^ s^−1^) of photosystem II were measured using the fluorometer (equipped with the Li-6800). The Φ_PSII_ was calculated from 1-(F_s_/F_ml_), where F_s_ is the minimum fluorescence emission and F_ml_ is the maximum fluorescence emission under steady-state illumination.

#### Damage assessment and multispectral images

The degree of damage to each shrub in the two plots was assessed by analysing photographs taken of each shrub three times during the experiment (18 June 2024, 1 and 29 August 2024). Six damage classes were defined based on the proportion of brown leaves (damage class 1: healthy plants, class 2: up to 20%, class 3: > 20% to 50%, and class 4: > 50% to 100% brown leaves, class 5: complete dieback of the aboveground shoot but resprouting of basal lateral shoots, class 6: complete die-off).

In addition, images were taken with a multispectral camera (P4 multispectral imaging system, DJI, China) on the same days as the damage assessment. We recorded the Normalised Difference Vegetation Index (NDVI) based on red (650 nm) and near-infrared (840 nm) images, which were then analysed for differences in NDVI between the two plots.

#### Biomass

At the end of the growing season (23 September 2024), all shrubs were harvested, and branches, leaves, and roots were separated and oven-dried at 80 °C for at least 2 days. Each part was then weighed to determine the dry mass. As the dwarf shrubs were harvested after the end of the growing season, the leaf biomass could only be determined for the evergreen species (*A. uva-ursi*, *V. vitis-idaea*, *C. vulgaris* and *S. officinalis*). In Plot10, *C. vulgaris* did not survive the drought and therefore the leaf biomass could not be determined.

### Statistical analysis

Statistical analyses and multispectral images processing were performed using R software (R Core Team 2023). One-Way Analysis of Variance (ANOVA) followed by the Tukey post-hoc test was used to detect differences within each species (seasonal changes in the damage levels and predawn *versus* midday). Normality and homoscedasticity assumptions were tested prior to analysis using the Shapiro–Wilk Normality Test and the Fligner-Killeen Test of Homogeneity of Variances, respectively, and if these assumptions were not met, the Kruskal–Wallis Test was used instead. For the biomass data, we additionally performed a two-way ANOVA with species and substrate depth as fixed factors, including their interaction, to test for species-specific responses to substrate depth. When assumptions of normality and homoscedasticity were not met, data were log-transformed prior to analysis. Post-hoc comparisons were carried out using Tukey post-hoc test. For pairwise comparisons of NDVI values of each species between the 10 and 30 cm plots, the pairwise t-test was used, and when the assumptions of normality and homoscedasticity were not met, the non-parametric Wilcoxon test was used instead. Multispectral images were processed in R to extract the pixel values from the center of each shrub’s crown in the two plots. In addition to functions from the package “stats” (R Core Team 2023), data.table (Dowle and Srinivasan 2023), stringr (Wickham 2022), ggplot2 (Wickham 2016), and ggpubr (Kassambara 2023) were used for organizing, filtering and visualizing data. Values are given as mean ± standard error (SE).

## Results

### Climatic conditions

**Fig. 1:**
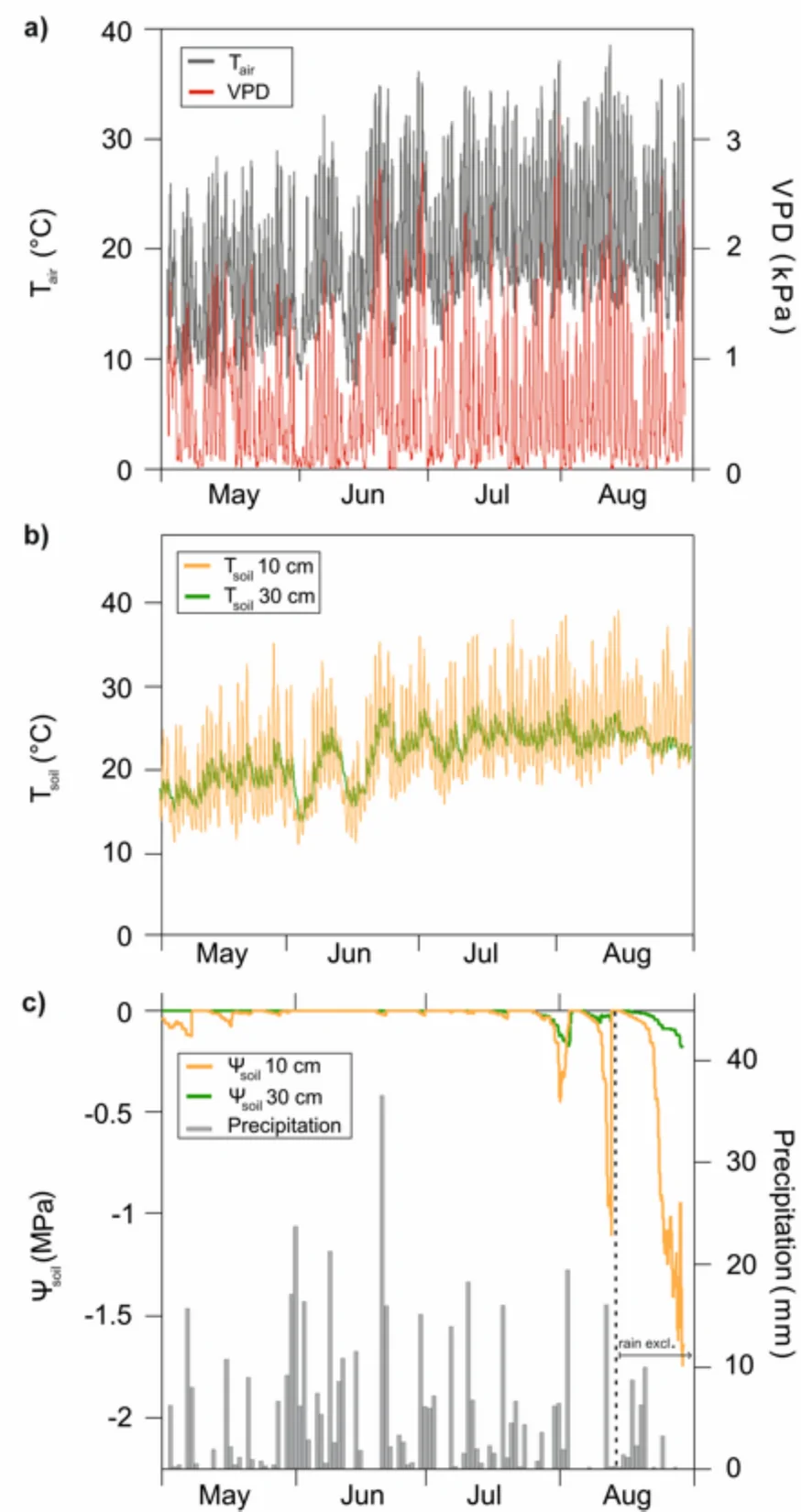
**a)** Air temperature (black line; T_air_) and vapour pressure deficit (red line; VPD), **b)** soil temperature (T_soil_), **c)** soil water potential (Ψ_soil_) in the 10 cm substrate (orange lines) and in the 30 cm substrate (green lines) and precipitation (grey bars) during the experiment. The dotted line in **c)** represents the onset of the “rain exclusion” period, when precipitation was excluded from both plots (for details see Materials and Methods)

During the experiment, T_air_ averaged 19.1 ± 0.05°C. VPD averaged 0.64 ± 0.01, 0.68 ± 0.01, 0.71 ± 0.01 and 0.69 ± 0.01 in May, June, July and August, respectively, with several peaks above 3 kPa during the summer (Fig. 1a). The daily maximum T_soil_ was higher in the 10 than in the 30 cm plot throughout the growing season (Fig. 1b). Mean T_soil_ was 20.7 ± 0.07 and 19.5 ± 0.06 °C and the maximum T_soil_ was 39.2°C (mid-August) and 28.0°C (end of July) in the 10 and in the 30 cm plot, respectively. The total rainfall was 467 mm (Fig. 1c).

Ψ_soil_ was rather constant (with minor decreases in the 10 cm plot in May) at around 0 MPa for both plots until the end of July, due to repeated precipitation (Fig. 1c). At the end of July, Ψ_soil_ became more negative and reached −0.45 and −0.17 MPa in the 10 and in the 30 cm plot, respectively, on 31 July. In early August, there was a second reduction in the shallower substrate, approaching −1.1 MPa, while the soil in the deeper substrate still showed moderate Ψ_soil_. In the following dry period (from 13 to 30 August 2024 both plots were covered; see Plant material and study design) the 10 cm plot dried to −1.75 and the 30 cm substrate to −0.24 MPa.

### Gas exchange and water relations during season

**Fig. 2:**
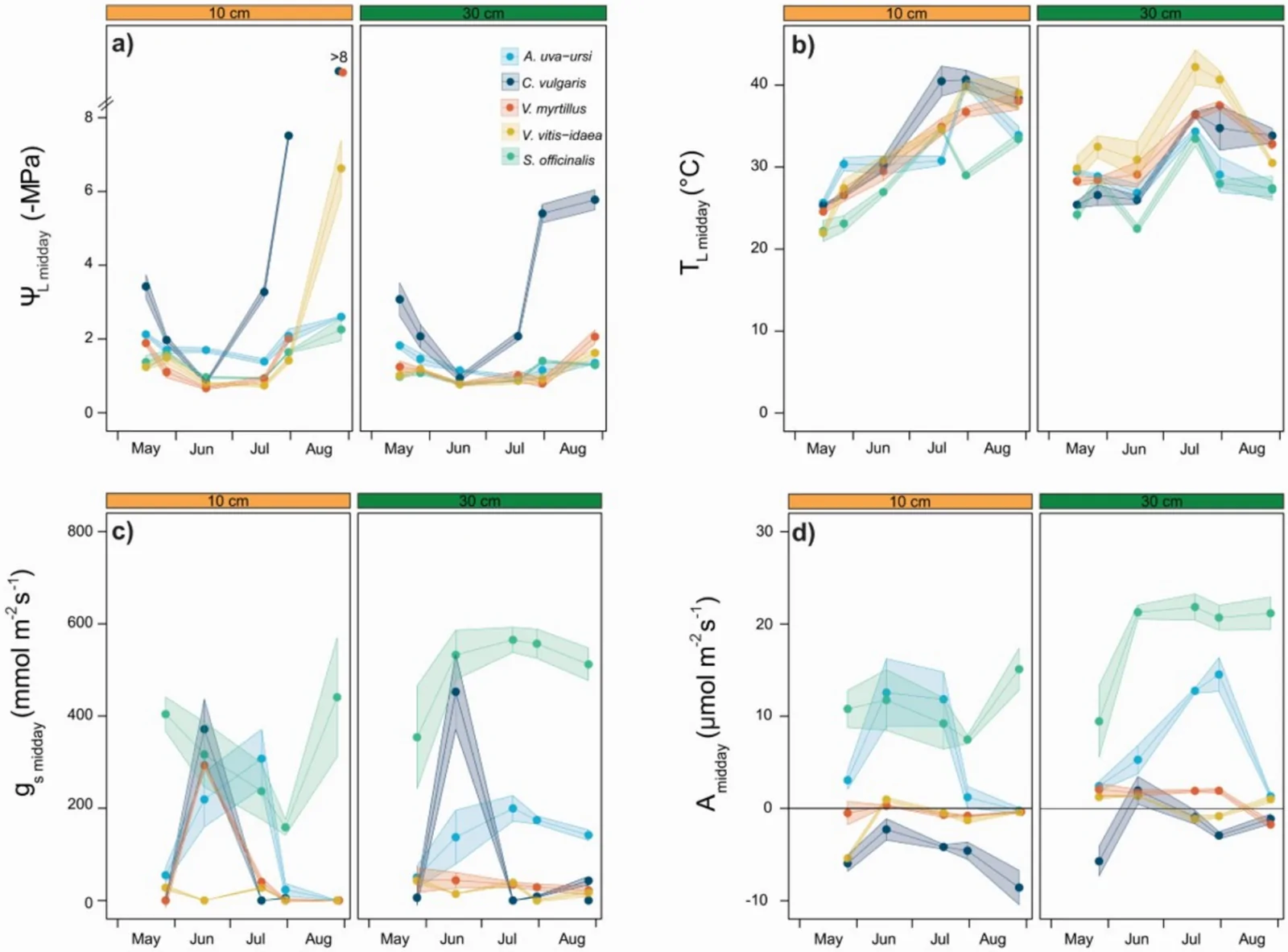
Seasonal course of **a)** midday leaf water potential (Ψ_L midday_; MPa), **b)** leaf temperature (T_L midday_; °C), **c)** stomatal conductance (g_s midday_; mmol m^−2^ s^−1^), and **d)** photosynthetic rate (A_midday_; µmol m^−2^ s^−1^) measured at midday during sunny days in the 10 cm substrate (10 cm) and in the 30 cm substrate (30 cm) plot. Ψ_L midday_ and T_L midday_ monitoring started in mid and g_s midday_ and A_midday_ at the end of May. Mean ± SE per species. Note that in **a)**, the Ψ_L midday_ of *C. vulgaris* and *V. myrtillus* fell below the measurement limit of −8 MPa

Ψ_L midday_ was more negative in the 10 than in the 30 cm plot for all measurements (Fig. 2a), with means of −2.31 ± 0.31 MPa and −1.54 ± 0.17 MPa, respectively, including all species and the whole season. The least negative Ψ_L midday_ occurred in mid-June for *V. myrtillus*, while the most negative Ψ_L midday_ was measured in *C. vulgaris* and *V. myrtillus* in August, when it even dropped below −8 MPa in the 10 cm substrate. T_L midday_ was generally higher in the 10 than in the 30 cm plot, with a mean of 31.5 ± 0.81 and 30.7 ± 0.95°C, respectively, for all species over the whole season (Fig. 2b). T_L midday_ increased until July in both plots and was highest in *V. vitis-idaea* in mid-July, approaching 42.2°C in the deeper plot.

g_s midday_ was higher in the 30 cm than in the 10 cm plot, with mean values of 169 ± 38.6 and 119 ± 27 mmol m^−2^ s^−1^, respectively, for all species over the whole season (Fig. 2c). However, species differed markedly in their stomatal behavior. The maximum g_s midday_ was measured in *S. officinalis* in the 30 cm plot (565 ± 27.2 mmol m^−2^s^−1^) on 18 July 2024, followed by *A. uva-ursi* in the 10 cm plot (307 ± 63.5 mmol m^−2^s^−1^) on the same date. The two *Vaccinium* species closed their stomata in both plots at moderately negative Ψ_soil_ (*i.e*., before the end of July) and *C. vulgaris* showed low g_s midday_, except in mid-June. Midday photosynthetic rate (A_midday_, µmol m^−2^ s^−1^) was higher for all shrubs in the 30 than in the 10 cm plot, with mean values of 4.81 ± 1.61 and 2.32 ± 1.18 µmol m^−2^ s^−1^, respectively, over the whole season (Fig. 2d). The highest values of A_midday_ were recorded for *S. officinalis* at the end of July, with 21.85 ± 1.41 µmol m^−2^s^−1^ in the 30 cm plot, followed by *A.* *uva-ursi* reaching 14.52 ± 1.83 (end of July) and 12.75 ± 0.27 (mid-June) µmol m^−2^ s^−1^ in the 30 and 10 cm plot, respectively.

Φ_PSII_ (Suppl. Fig. S1a) was slightly higher for all shrubs in the 30 (mean of 0.17 ± 0.02) than in the 10 cm plot (mean of 0.15 ± 0.02). The highest values were recorded for *S. officinalis* at the end of July, with a mean of 0.43 ± 0.02 and 0.49 ± 0.01 in the 10 and in the 30 cm plot, respectively. Also, *A. uva-ursi* showed high Φ_PSII_, but it decreased sharply at the end of July in the 10 cm plot. *Vaccinium* species and *C. vulgaris* showed the lowest Φ_PSII_ in both plots. ETR (Suppl. Fig. S1b) was higher for all shrubs in the 30 (mean of 77.64 ± 9.15 µmol m^−2^ s^−1^) than in the 10 cm plot (mean of 70.61 ± 9.27 µmol m^−2^ s^−1^). The highest ETR values were measured for *S. officinalis* and *A. uva-ursi*, while the lowest were recorded for *Vaccinium* species and *C. vulgaris*. In the 10 cm substrate, there was a sharp decrease in ETR of *A. uva-ursi* from mid- to late July.

### Gas exchange and water relations during the humid period

During the humid period (in mid-July, see Materials and Methods), T_air_ ranged from 15.9°C in the morning to a maximum of 32.1°C in the afternoon, Ψ_soil_ was close to 0 MPa in both plots. Average surface substrate temperature at midday was 52.1 ± 1.46 and 47.9 ± 2.48°C in the 10 and in the 30 cm plot, respectively. The average daily maximum PAR was 1771 ± 14 µmol m^−2^ s^−1^.

**Fig. 3:**
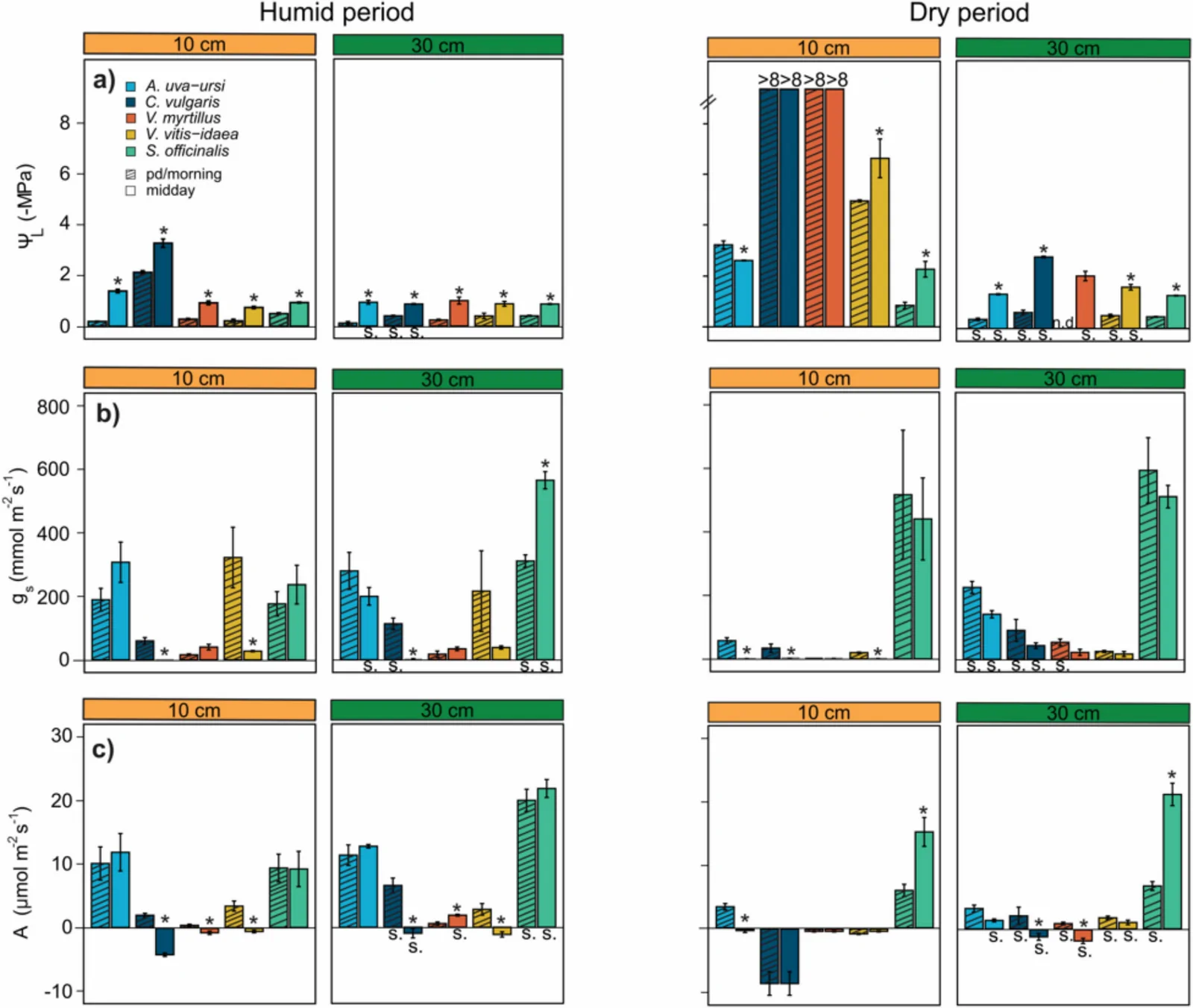
**a)** Leaf water potential (Ψ_L_; MPa), **b)** stomatal conductance (g_s_; mmol m^−2^s^−1^), and **c)** photosynthetic rate (A; µmol m^−2^ s^−1^), measured at pre-dawn (Ψ_L_) and morning (A and g_s_; see Materials and Methods) and at midday during the humid and the dry period in the 10 cm substrate (10 cm) and in the 30 cm substrate (30 cm). Asterisks indicate significant differences between predawn and midday values and “s.” indicates significant differences between the two plots. No Ψ_L midday_ values are available for *V. myrtillus* in the deeper substrate during the dry period (“n.d.”). Mean ± SE. Note that in **a)**, the Ψ_L pd_ and Ψ_L midday_ of *C. vulgaris* and *V. myrtillus* in the 10 cm plot fell below the measurement limit of −8 MPa

**Fig. 4:**
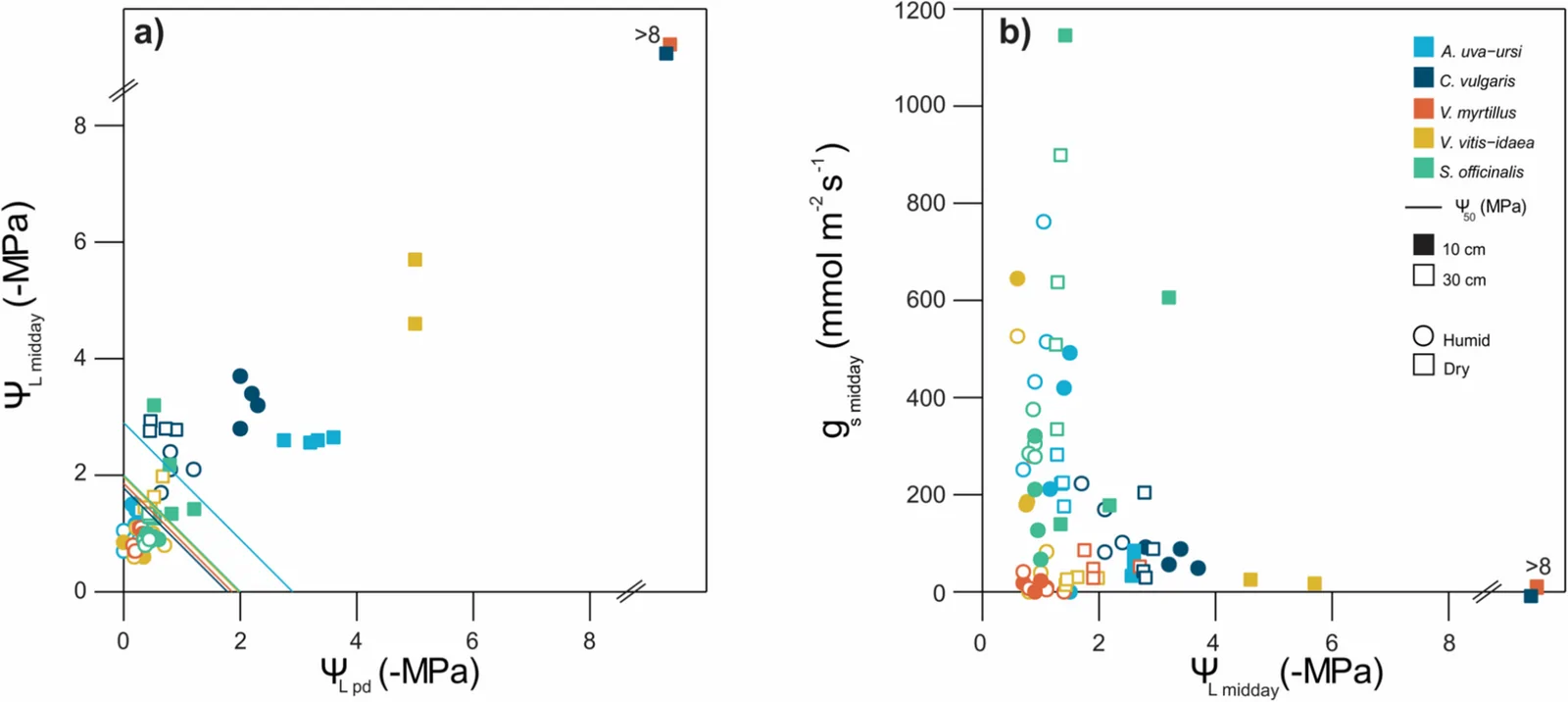
Correlation between **a)** predawn water potential (Ψ_L pd_; MPa) and midday water potential (Ψ_L midday_; MPa), and **b)** Ψ_L midday_ and stomatal conductance at midday (g_s midday_; mmol m^−2^ s^−1^), during humid (circles) and dry (squares) periods. Filled symbols represent the 10 cm substrate (10 cm), open symbols the 30 cm substrate (30 cm). In **a)**, colored lines indicate the threshold of 50% loss of conductivity (Ψ_50_; MPa) for each species

Ψ_L_ (Fig. 3a) was close to 0 MPa at pre-dawn and significantly decreased at midday to −1.35 ± 0.18 and −0.85 ± 0.06 MPa (average of all species) in the 10 and in the 30 cm plot. In the 10 cm plot, *C. vulgaris* had the lowest Ψ_L pd_ (−2.13 ± 0.06 MPa) and Ψ_L midday_ (−3.28 ± 0.16 MPa), whereas in the 30 cm substrate all species had similar Ψ_L midday_ and Ψ_L pd_ (Figs. 3 and 4a). Mean g_s_ at midday for all species were higher in the deeper (148.41 ± 41.11 mmol m^−2^ s^−1^) than in the shallower plot (119.29 ± 38.8 mmol m^−2^ s^−1^; Fig. 3b). At midday, *C. vulgaris* and *Vaccinium* species had lower g_s_ than the other shrubs, and *S. officinalis* had high g_s_ in both plots (Fig. 4b).

**Fig. 5:**
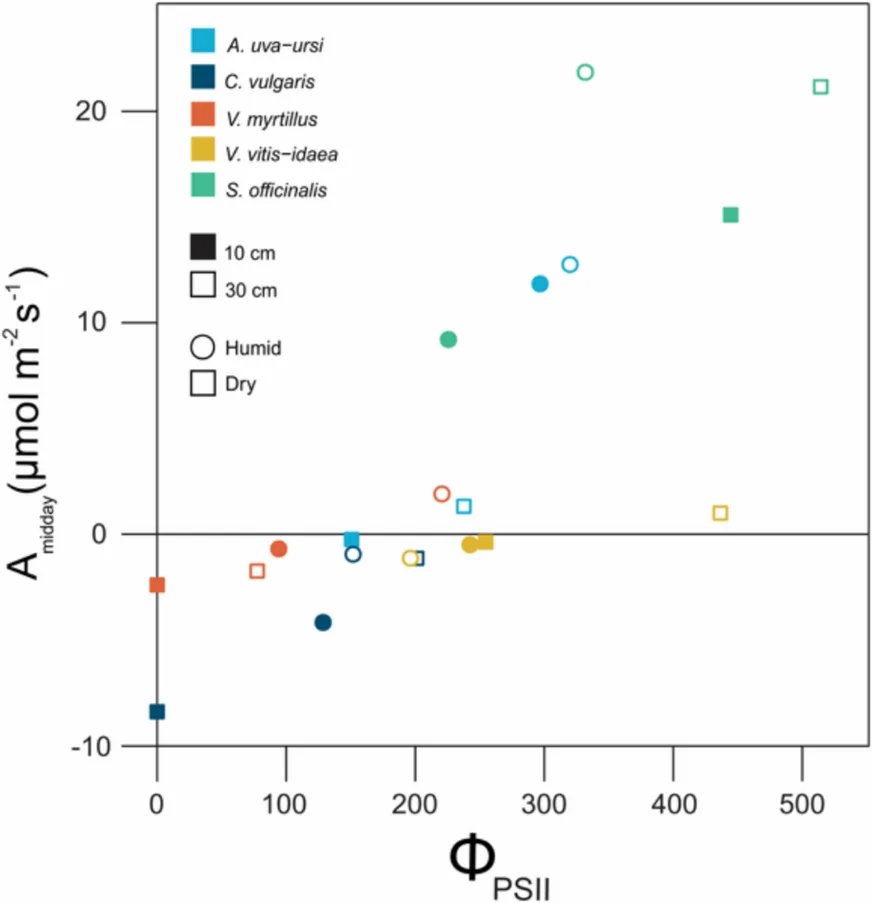
Correlation between the quantum yield of photosystem II (Φ_PSII_) and the net photosynthesis at midday (A_midday_; µmol m^−2^ s^−1^) during the humid (circles) and dry (squares) periods in the 10 cm substrate (filled symbols) and in the 30 cm substrate (open symbols)

Mean A at midday for all species was higher in the 30 cm (6.08 ± 2.47 µmol m^−2^ s^−1^) than in the 10 cm plot (4.82 ± 2.01 µmol m^−2^ s^−1^; Figs. 3c and 5). *Vaccinium* species and *C. vulgaris* exhibited overall low A_midday_*. S. officinalis* and *V. myrtillus* showed higher A in the 30 than in the 10 cm plot.

### Gas exchange and water relations during the dry period

During the dry period (sunny days with low soil water content, due to rain exclusion), T_air_ ranged from 18.7°C to a maximum of 26.6°C in the afternoon. Ψ_soil_ was on average −1.45 ± 0.03 and −0.15 ± 0.003 MPa in the 10 and in the 30 cm plot, respectively. Average surface substrate temperature at midday was 48.69 ± 3.54°C and 35.21 ± 4.24°C in the 10 and in the 30 cm plot, respectively. The average daily maximum PAR was 1903 ± 52 µmol m^−2^ s^−1^.

For *V. myrtillus* and *C. vulgaris,* both Ψ_L pd_ and Ψ_L midday_ during the drought fell below −8 MPa in the 10 cm plot (Figs. 3 and 4a) while Ψ_soil_ at that time was −1.75 MPa (Suppl. Fig. S2), strongly indicating that they did not survive the drought, while all shrubs survived in the 30 cm plot. The mean Ψ decreased from predawn (Ψ_L pd_ −4.6 ± 1.1 MPa in the 10 cm and −1.06 ± 0.5 MPa in the 30 cm plot; Fig. 3a) to midday (Ψ_L midday_ −5.01 ± 1 MPa and −1.8 ± 0.3 MPa in the 10 and in the 30 cm plot, respectively). For most species, a significant decrease in Ψ_L_ between predawn and midday was observed, and Ψ_L pd_ was close to Ψ_soil_ in the 30 cm plot but distinctly more negative in the 10 cm plot for all shrubs except for *S. officinalis* (Fig. 3, Suppl. Fig. S2 and Fig. 4a).

Overall, g_s_ was lower than in the humid period, except for *S. officinalis* (Fig. 3b). Surviving shrubs in the 10 cm plot tended to have lower g_s_ than those in the 30 cm substrate (Fig. 4b). For all species, A decreased from the humid to the dry period (Fig. 3c and 5). Species in the 10 cm plot tended to have lower A than those in the 30 cm plot.

### Damage assessment and NDVI images

**Fig. 6:**
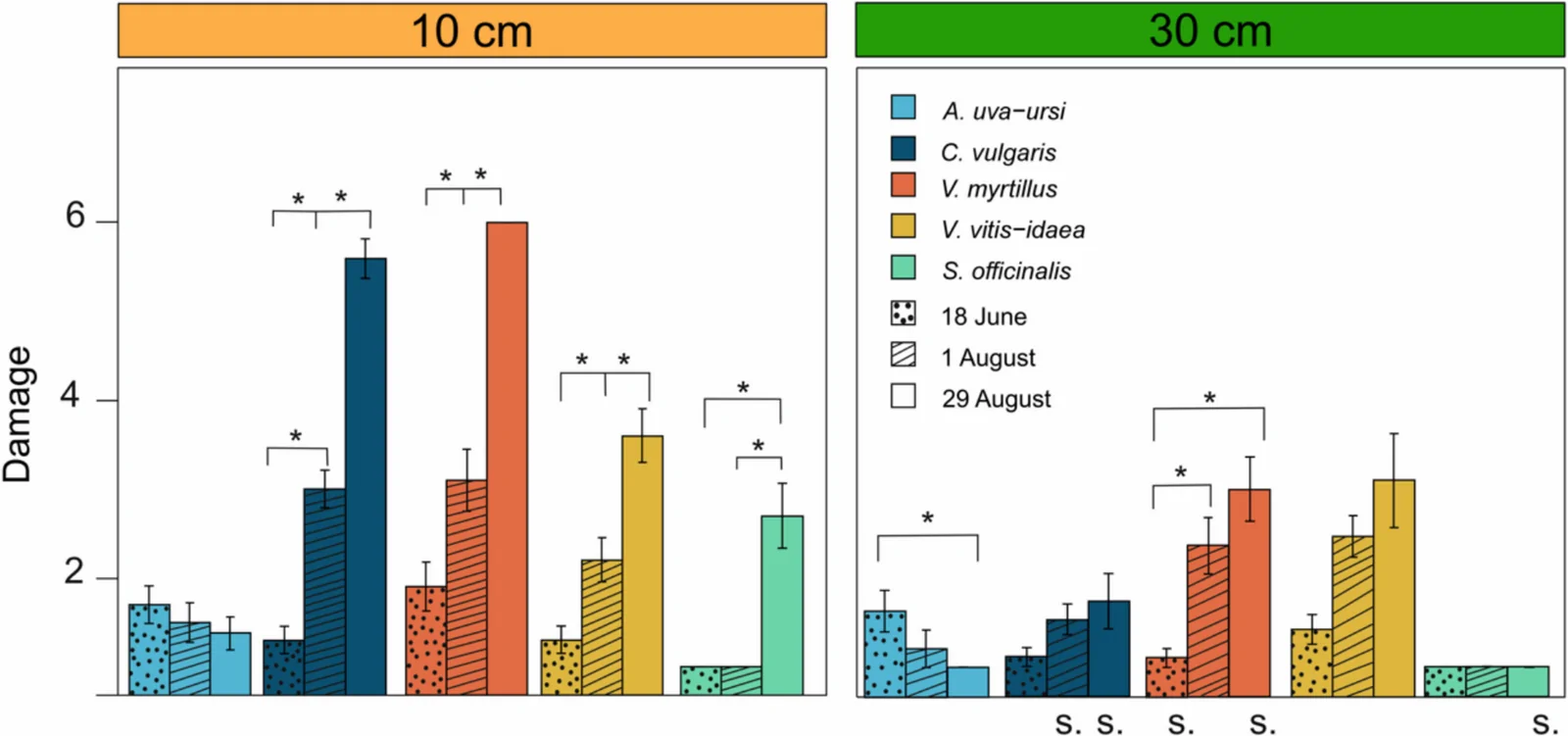
Damage classes (1: 0% brown leaves, 2: 1 to 20% brown leaves, 3: 21 to 50% brown leaves, 4: 51 to 99% brown leaves, 5: die-back but resprouting, 6: died plant) determined at three time points (mid-June, early and end of August) in the 10 cm substrate (10 cm) and in the 30 cm substrate (30 cm). The assignment of the shrubs to the damage classes was based on the examination of photographs. “s.” indicates significant differences between plots, while asterisks indicate significant differences between dates. Mean ± SE

Overall, the level of damage increased during the growing season for all plants in both plots (Fig. 6). A lack of damage was only observed in *S. officinalis* in the 30 cm plot. *C. vulgaris* and *V. myrtillus* showed the highest damage of all species in the 10 cm plot, reaching damage classes 3 at the beginning and 6 at the end of August. These species did not survive the intense drought in August. *V. vitis-idaea* showed signs of progressively increasing damage during the growing season in both plots, while *A. uva-ursi* and *S. officinalis* were relatively healthy throughout the season. In the 30 cm substrate, all plants remained below the damage class 2 in mid-June and below class 3 in early and late August.

**Fig. 7:**
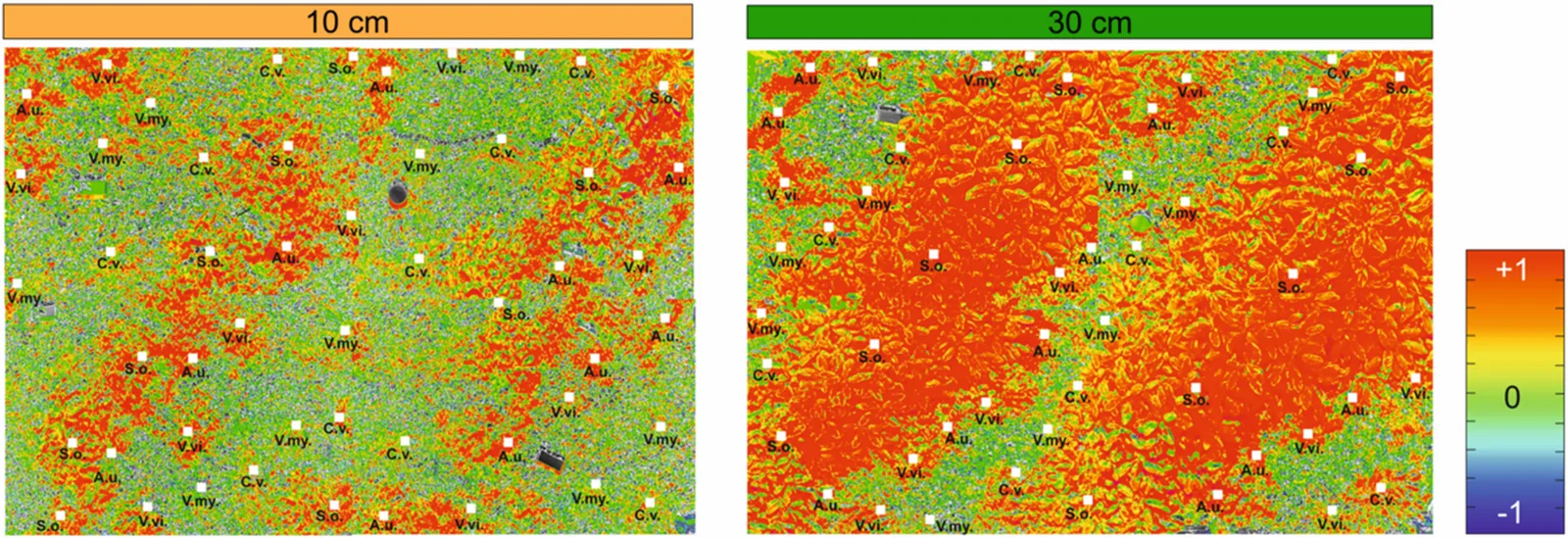
NDVI images of the 10 cm substrate (10 cm) and the 30 cm substrate (30 cm) taken by a multispectral camera at the end of August. Red and green colours indicate higher (*i.e.*, greater biomass and chlorophyll content) and lower (*i.e.*, lower biomass and chlorophyll content) NDVI values, respectively. The white squares show the centre of the crown of the shrubs and refer to the following species: A.u. is *A. uva-ursi*, C. v. is *C. vulgaris*, V.my. is *V. myrtillus*, V.vi. is *V. vitis-idaea*, and S.o. is *S. officinalis*

The multispectral images indicated similar (not significantly different, *p*-value = 0.46) NDVI values for the two plots in mid-June, with shrubs in the 30 cm plot showing a mean NDVI of 0.664 ± 0.022 and shrubs in the 10 cm plot a mean NDVI of 0.686 ± 0.022 (Suppl. Fig. S3). The plots started to differentiate in early August, when *S. officinalis* had evidently grown in the 30 cm substrate more than in the 10 cm one, and other shrubs such as *C. vulgaris* and *V. myrtillus* started to show drought symptoms in the shallower plot. By the end of the growing season, the differences between plots had become even more pronounced, with *S. officinalis* shrubs leading to large patches with high NDVI in the deeper plot (Fig. 7). At this stage, the mean NDVI was 0.725 ± 0.018 for shrubs in the 30 cm and 0.566 ± 0.019 for shrubs in the 10 cm plot (*p*-value = 6.38*10^–6^).

### Biomass

**Fig. 8:**
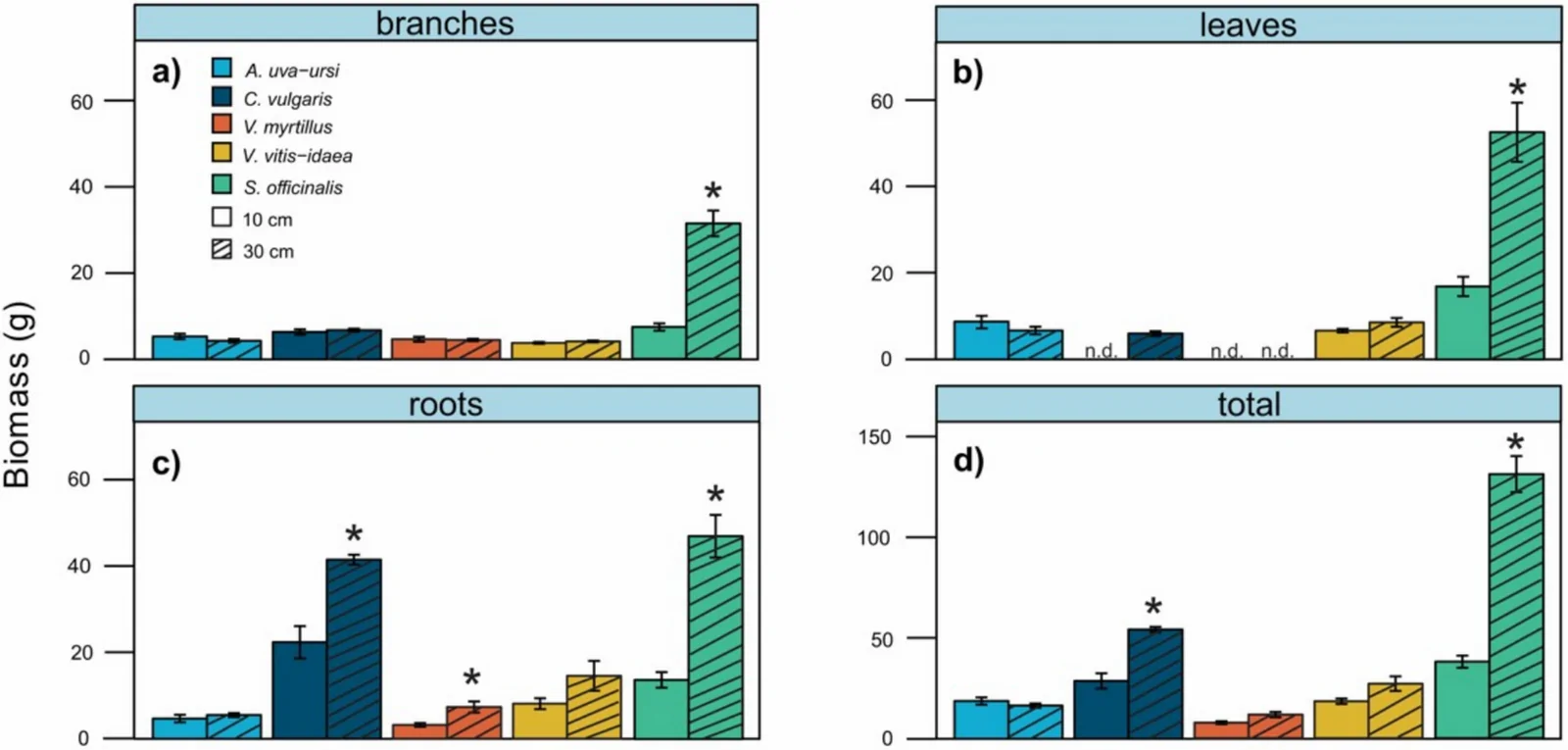
Dry weight (Biomass, g) at the end of the experiment of **a)** branches, **b)** leaves, **c)** roots, and **d)** their sum (total) in the 10 cm substrate (10 cm) and in the 30 cm substrate (30 cm). Asterisks indicate statistically significant differences among species within a substrate, “n.d.” indicates missing data as shrubs shed their leaves. Mean ± SE. Note the different scale in **d)**

Leaves, branches, and roots were analysed separately to determine their biomass (Fig. 8). In the 10 cm plot, *C. vulgaris* and *V. myrtillus* did not survive the drought, so only the remaining branches and roots were measured, whereas in the 30 cm plot all shrubs survived. At the end of the season,* S. officinalis* had a significantly higher biomass of all the three plant parts measured in the 30 cm when compared to the 10 cm plot. *V. myrtillus* had a significantly higher biomass of branches and roots and *C. vulgaris* had a significantly higher biomass of roots in the 30 cm than in the 10 cm plot. In the 10 cm substrate, *S. officinalis* had the highest leaf biomass (mean of 16.92 ± 2.38 g), and *C. vulgaris* the highest root biomass (mean of 22.38 ± 3.95 g) among species. In the 30 cm substrate, *S. officinalis* had the highest biomass in all three plant organs (mean of 31.38 ± 3.18 g for branches, 52.68 ± 7.23 g for leaves, and 47.02 ± 5.24 g for roots) among species, followed by *A. uva-ursi* (mean of 4.31 ± 0.43 g for branches, 6.69 ± 0.92 g for leaves, and 5.50 ± 0.50 g for roots).

## Discussion

This experiment examined the response to drought stress of four Alpine dwarf shrubs (*Arctostaphylos uva-ursi*, *Vaccinium myrtillus*, *V. vitis-idaea*, *Calluna vulgaris*) and the Mediterranean shrub *Salvia officinalis* for comparison under green roof conditions with different substrate depths. The temperature changes were less extreme, and the soil water potential remained higher in the 30 cm plot, resulting in lower drought stress compared to the 10 cm plot. At the peak of the drought, all shrub species survived in the 30 cm plot, whereas *V. myrtillus* and *C. vulgaris* died in the 10 cm plot (indicated by very low plant water potentials, negative photosynthetic rates, complete leaf shedding and dry branches). Drought effects on vitality and growth were species-specific and more pronounced in the shallower substrate. In the following, we discuss the differences in environmental conditions between the 10 cm and the 30 cm plot and their effects on physiological responses of the five study species.

The vegetation period 2024 was comparably humid, and temperatures were moderate (Fig. 1), which was overall favourable for the planted shrubs. However, even before the onset of the dry period (end of August; rain exclusion period in Fig. 1c), environmental conditions of the 10 and 30 cm plot differed: Ψ_soil_ was slightly more negative in the 10 than in the 30 cm plot on several days. In a Mediterranean climate, Savi et al. (2015) also found lower soil water potentials (around −2.2 MPa) in shallower substrates (10 cm depth) than in deeper ones (ca. −1 MPa and 13 cm depth) at the beginning of the summer dry period. In line with previous studies in semi-arid and temperate climates (Gößner et al. 2025; Eksi et al. 2017; Reyes et al. 2016), the mean daily T_soil_ was similar in both plots (around 21 °C), while the amplitude of daily temperatures and maximum T_soil_ were substantially higher in the 10 cm than the 30 cm plot. These differences between shallower and deeper plots increased remarkably during the dry period, when a Ψ_soil_ of −1.75 MPa was reached in the former, while it was only −0.20 MPa in the latter. Ψ_soil_ in the 10 cm plot thus was below the permanent wilting point (−1.5 MPa); as found on green roofs in Mediterranean areas (Tomasella et al. 2022) at the beginning of July and, more generally, during warm rainless periods in between rainy days.

At the peak of the drought, maximum T_soil_ in the 10 cm substrate was 39.2°C, compared to 26.6°C in the 30 cm substrate. While climatic conditions might have changed since earlier studies, these values still fall within the ranges reported for substrates of similar depths during warm periods. For instance, Eksi et al. (2017) observed maximum soil temperatures of ca. 35°C and 29 °C for substrate depths of 5 cm and 20 cm, respectively, in an extensive roof in a temperate climate in July. According to Savi et al. (2016a, b), a difference of only 3 cm in substrate depth can significantly affect maximum soil temperatures. In their study, they measured maximum temperatures of 44°C and 39.2°C in substrates of 10 cm and 13 cm, respectively. It is important to note that ongoing climate change may shift these thresholds in the future.

We also observed relevant higher leaf temperatures in the 10 cm (up to 41 °C, see Fig. 2b) compared to the 30 cm plot, especially during the dry period. Such temperatures are very close to the appearance of substantial cellular damage in the alpine shrub *V. gaultherioides*, which has a LT_50_ of 45 °C (lethal temperature for 50% of leaf cells; (Karadar et al. 2018). Observed damage in the presented experiment might thus have been caused, besides drought, also by heat stress. Notably, T_L midday_ varied remarkably among species (up to 10 °C difference), probably due to species-specific variation in stomatal regulation and thus transpirational cooling. Species with higher g_s midday_, such as *S. officinalis* (Fig. 2b), generally maintained lower T_L midday_. In contrast, species with stricter stomatal control, including *C. vulgaris* and *Vaccinium* species, exhibited limited evaporative cooling and reached the highest T_L midday_ values. Accordingly, Blanusa et al. (2013) found that the level of irrigation influences stomatal conductance and, consequently, the cooling “service” provided by the leaves (*e.g.*, well-watered plants with higher stomatal conductance exhibited leaf temperatures that were 2.5°C lower than those of water-stressed plants). However, this also depends on the species, which exhibit differences in stomatal conductance and therefore in leaf temperature. Monteiro et al. (2016) reported that leaf traits such as leaf colour, thickness, and pubescence also contribute to leaf temperature regulation on green roofs (*e.g.*, light-coloured and pubescent leaves with longer hairs were associated with lower leaf temperatures), again highlighting species-specificity in leaf cooling control on green roofs. Stomatal regulation on green roofs thus influences leaf temperature and directly affects growth through its role in photosynthesis.

The stomatal behaviour of the shrub species also corresponded to their photosynthetic performance, which directly affects growth through carbon assimilation. Species such as *S. officinalis* and *A. uva-ursi* characterised by high g_s midday_ displayed greater A_midday_. In contrast, *C. vulgaris* and *Vaccinium* species showed low g_s midday_ and negative A_midday_, particularly in the 10 cm plot and under drought stress. Besides stomatal limitation, A_midday_ in some study species was also reduced by limitation of the photosynthetic apparatus as indicated by reduced Φ_PSII_ and ETR during drought (Suppl. Fig. S1), most pronounced in *A. uva-ursi* (Fig. 5). This interpretation is supported by the linear relationship between A_midday_ and Φ_PSII_ (Fig. 5), indicating that drought-induced reductions in photosynthetic rates were linked to photochemical impairment. This is consistent with the study by Yu et al. (2015) on *Vaccinium corymbosum*, which reported both Φ_PSII_ and ETR to decrease with increasing water stress. Their study demonstrated that *V. corymbosum* re-irrigated after three weeks of drought showed full recovery, whereas plants re-irrigated after five weeks did not regain their photosynthetic capacity. On green roofs, the duration of drought periods (and their buffering by moisture in the substrate) might thus be decisive for the establishment of plants. Vice versa, successful species on the green roof will influence these buffers, with the water use strategies of plants determining transpirational losses.

In this study, stomatal regulation was similar in all analysed species, with stomata closing before a plant water potential of −2 MPa was reached (Fig. 4b). Remarkably high g_s_ values were observed in *S. officinalis*, and, to a lesser extent, in *V. vitis-idaea* and *A. uva-ursi*, indicating a more anisohydric behaviour, as also reported by Raimondo et al. (2015) for *S. officinalis*. Conversely, *C. vulgaris* and *V. myrtillus* had extremely low g_s midday_, and probably were the most isohydric species. Further analyses on the rooting system, water storage capacity and hydraulic segmentation (see also Savi et al. (2016a, b) are required to better understand the response patterns of these shrubs to varying soil moisture and to varying vapour pressure deficit.

Over season, and especially in the drought period Ψ_soil_ and, in consequence, Ψ_L_ of all species declined with more pronounced reductions in the 10 cm than in the 30 cm plot (Fig. 2a). *C. vulgaris* exhibited the most negative Ψ_L_ in both plots, reaching below −8 MPa in the 10 cm (where the species ultimately died; see below) and −5.77 ± 0.27 MPa in the 30 cm plot. Similarly, *Vaccinium* species reached low Ψ_L_ in the 10 cm (−6.63 ± 0.76 MPa in *V. vitis-idaea;* below −8 MPa in *V. myrtillus*), while only ca. −2 MPa in the 30 cm plot. Ψ_L_ of *A. uva-ursi* dropped below −2 MPa in the 10 cm; in contrast, it remained around −1.4 MPa in the 30 cm plot (see Fig. 2a). With respect to vulnerability to drought induced embolism (Ganthaler and Mayr 2015, 2021), *C. vulgaris* and *V. myrtillus* surpassed their Ψ_50_ (−1.78 MPa and −1.86 MPa, respectively) on both plots (Fig. 4a). Ψ_L_ of *V. vitis-idea* was below Ψ_50_ (−1.97 MPa) in the 10 cm, while it was above in the 30 cm plot. *S. officinalis* and *A. uva-ursi* generally remained above their Ψ_50_ (−1.61 MPa and −2.91 MPa, respectively; Ganthaler and Mayr 2021; Savi et al. 2016a, b) in both plots. According to vulnerabilities and Ψ_L_ reached, we expect excessive embolism formation in *C. vulgaris* and *V. myrtillus*, some embolism in *V. vitis-idea*, but no hydraulic failure in *S. officinalis* and *A. uva-ursi*, which fits overall to observed damage patterns.

Drought (probably in combination with heat stress) caused overall higher damage in the 10 cm plot (Fig. 6), including complete defoliation of *C. vulgaris* and *V. myrtillus*, about 50% brown leaves in *V. vitis-idaea* and *S. officinalis* and less than 20% brown leaves in *A. uva-ursi*, but only the first two species experienced dieback. Some species maybe risk the loss of leaves and form new leaves after drought (Savi et al. 2016a, b; Raimondo et al. 2015).

Damage pattern together with gas exchange and water relations clearly demonstrate that the substrate depth had a major influence on plant performance, though differences between species were pronounced. This was further confirmed by NDVI analyses (Fig. 7), and by the two-way ANOVA, which showed significant effects of species, substrate depth and their interaction on biomass, indicating that the magnitude of the depth effect was species-specific (Suppl. Table S2). For example, *S. officinalis* and *C. vulgaris* did not only have the highest biomass but also showed the largest difference between the plots. Similarly, Zhang et al. (2014) reported that a 10 cm increase in substrate depth doubled shrub (*S. officinalis*, *Thymus serpyllum*, and *Thymus mongolicum*) survival on a green roof and also Savi et al. (2016a, b) demonstrated higher shrub survival on higher substrate (*e.g.*, *Cistus salviifolius*, *Emerus majus*, *Ligustrum vulgare*, *Phillyrea angustifolia*). In a green roof study in a Mediterranean climate, Ntoulas et al. (2013) found NDVI on a green roof planted with herbaceous species to be much higher on a 15 compared to 7.5 cm substrate layer. And an increased biomass production on higher substrate on green roofs in France and eastern North America was reported by Dusza et al. (2017) and Thuring et al. (2010). Thus, increased substrate depth as well as the selection of appropriate species (Du et al. 2019) are important tools to optimise plant establishment and vitality on green roofs.

## Conclusions

Several Alpine dwarf shrubs seem suitable for planting on green roofs in European temperate regions. Out of the four species tested, *A. uva-ursi and V. vitis-idaea* showed best performance under experimental conditions. These species were characterised by comparably high gas exchange rates during humid periods and moderate leaf water potentials under drought stress (sharing the strategy of *S. officinalis*), which resulted in a high vitality and good survival even with shallow substrate. In contrast, *C. vulgaris* and *V. myrtillus* could not maintain Ψ_L midday_ above the threshold for embolism formation despite reductions in g_s midday_, and their development was limited by very low gas exchange rates even under more favorable conditions. The comparison of shrubs growing on substrate layers of 10 and 30 cm in depth revealed much better plant performance (in terms of vitality and growth) for the latter. The deeper substrate had higher humidity during periods of drought, and it also buffered temperature extremes. From an ecological viewpoint, increased substrate depths on green roofs are thus desirable, as they will lead to greater plant biodiversity and may also enable the establishment of (Alpine) shrubs. However, the investments needed for building statics and green roof materials might not allow for higher substrate depths across the entire roof. Therefore, a promising and feasible solution for supporting shrub survival and accounting for their species-specific drought responses could be to use structured roofs with areas of low and high substrate depths.

## Supplementary Information

Below is the link to the electronic supplementary material.Supplementary file1 (PDF 807 KB)

## Data Availability

The datasets analysed during the current study are available from the corresponding author on reasonable request.

## Abbreviations

Ψ_L _ (MPa): Leaf water potential
Ψ_50 _ (MPa): Leaf water potential at 50% loss of conductivity
Ψ_soil _ (MPa): Soil water potential
Ψ_L__ midday _ (MPa): Leaf water potential at midday
Ψ_L__ pd _ (MPa): Leaf water potential at predawn
PAR (µmol m^−^^2^ s^−^^1^): Photosynthetically active radiation
T_air_: Air temperature
T_soil_ (°C): Soil temperature
T_L__ midday _ (°C): Leaf temperature at midday
LT_50 _ (°C): Lethal temperature for 50% of leaf cells
VPD (kPa): Vapour pressure deficit
A (µmol m^−^^2^ s^−^^1^): Net photosynthetic assimilation
g_s_ (mmol m^−^^2^s^−^^1^): Stomatal conductance
A_midday _ (µmol m^−^^2^ s^−^^1^): Net photosynthetic assimilation at midday
g_s__midday_ (mmol m^−^^2^s^−^^1^): Stomatal conductance at midday
Φ_PSII_: Quantum yield of photosystem II
ETR (µmol m^−^^2^ s^−^^1^): Electron transport rate
NDVI: Normalised Difference Vegetation Index
