# Supplementary material for: Alpine dwarf shrubs on green roofs: species-specific physiological responses to substrate depth and drought intensity
Source: Plant Soil. 2026 Jul 9;525(2):1465–80. doi: 10.1007/s11104-026-08836-6 (PMC13526063; doi:10.1007/s11104-026-08836-6)
Supplement: Supplementary file 1 [file 11104_2026_8836_MOESM1_ESM.pdf]

<u>**Supplementary Material**</u>

Alpine dwarf shrubs on green roofs: species-specific physiological responses to substrate depth and drought intensity

Carlotta Musso, Friederike Barkmann, Johannes Rüdisser, Stefan Mayr, Andrea Ganthaler

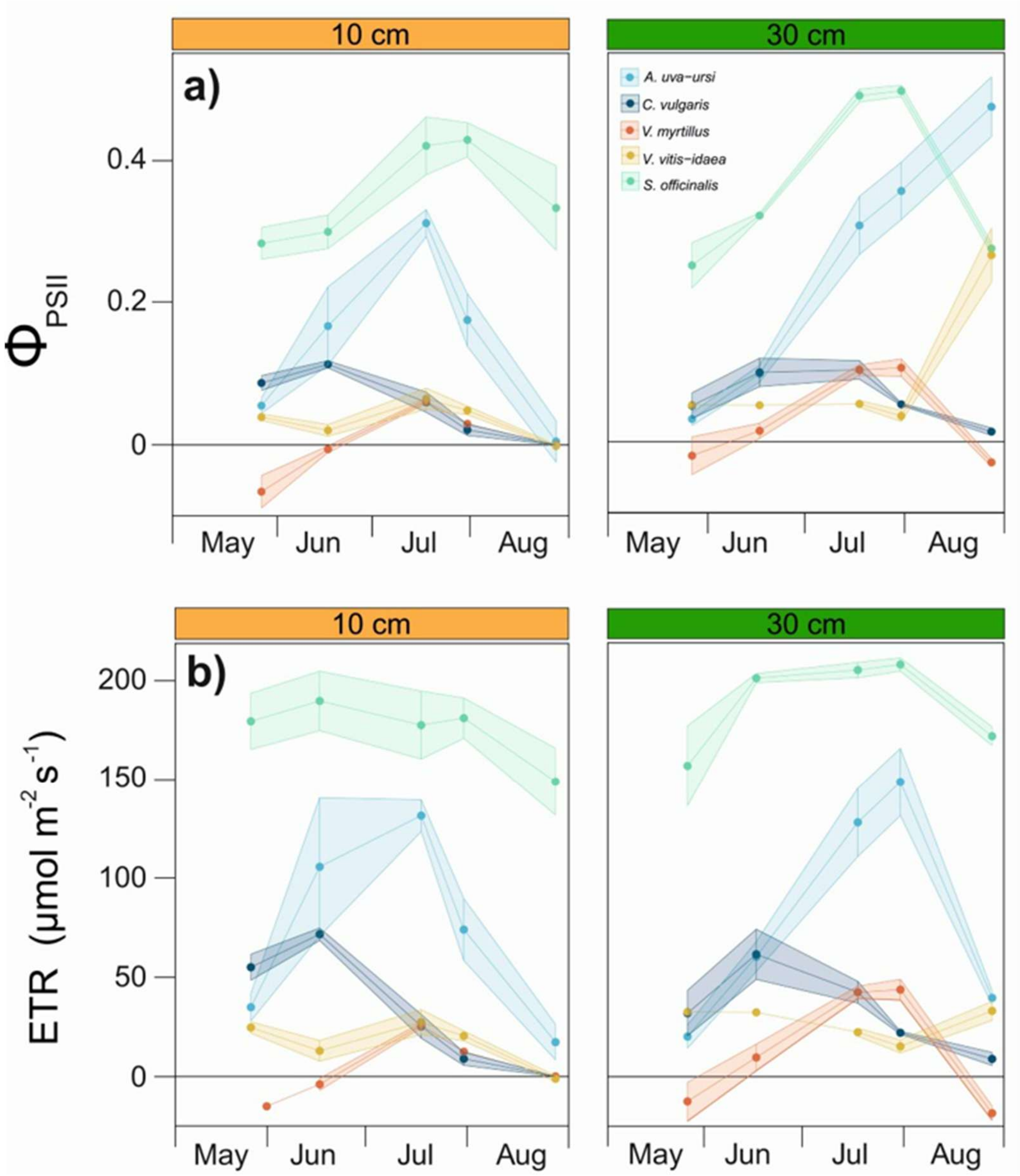

Fig. S1 Seasonal course of **a)** the quantum yield of photosystem II (Φ_{PSII}) and **b)** electron transport rate (ETR; µmol m^{-2} s^{-1}) measured at midday during sunny days in the 10 cm and in the 30 cm substrate plot. Mean ± SE per species.

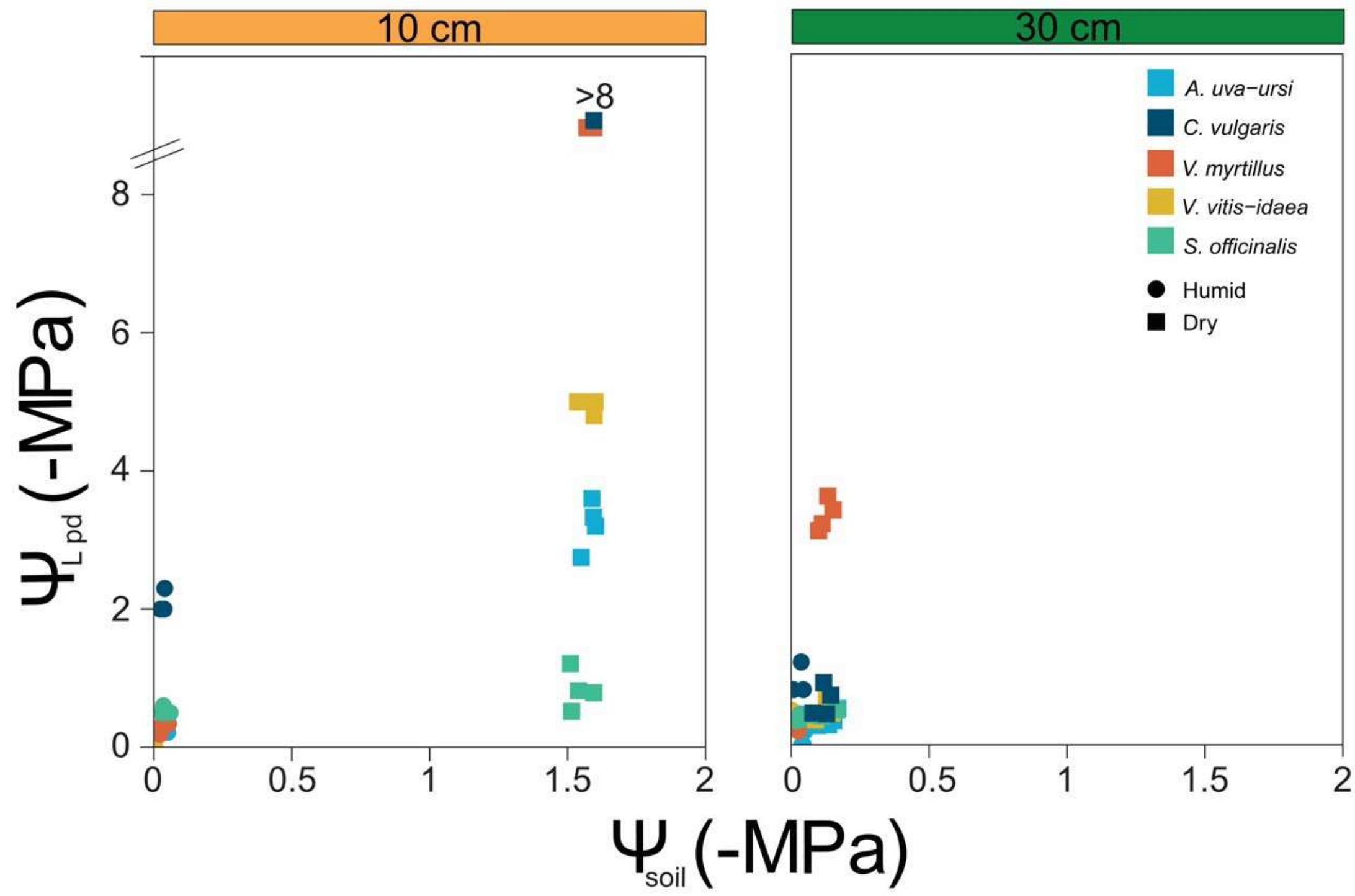

Fig. S2 Correlation between predawn water potential ($\Psi_{L\ pd}$; MPa) and soil water potential (Ψ_{soil}; MPa) during humid (Humid; circles) and dry (Dry; squares) periods in the 10 cm (10 cm) and in the 30 cm substrate (30 cm).

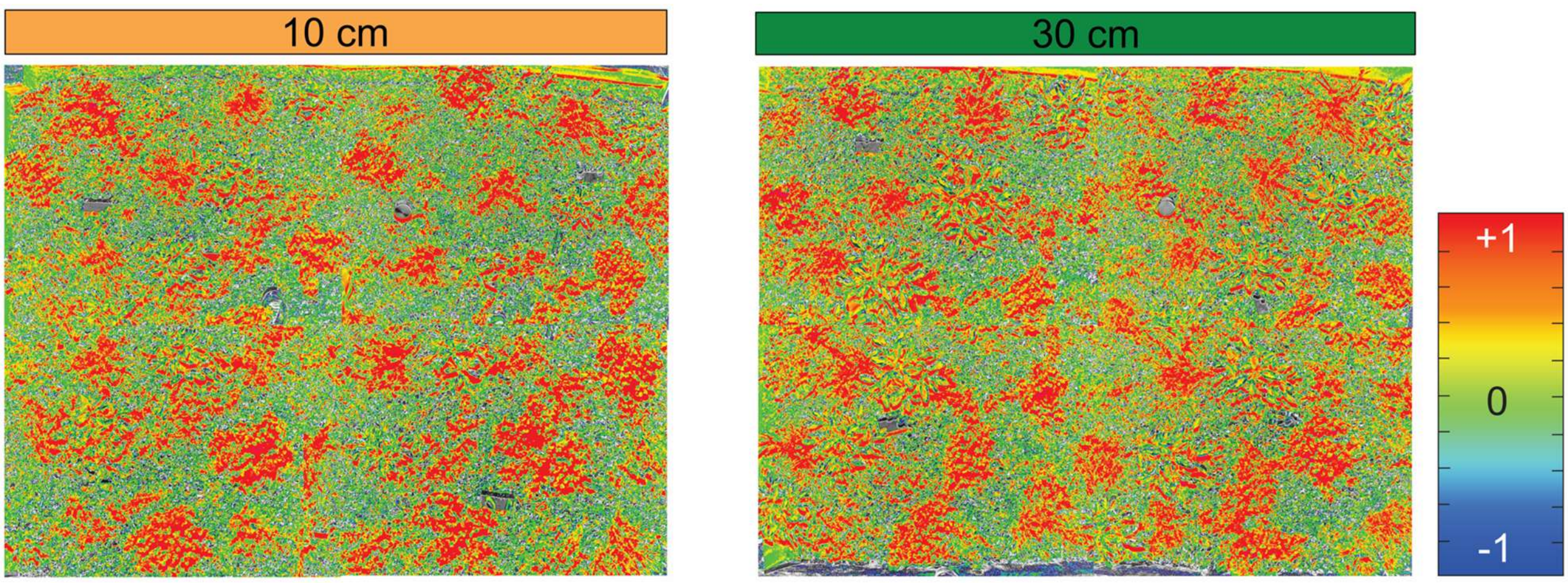

Fig. S3 NDVI images of the 10 cm substrate (10 cm) and the 30 cm substrate (30 cm) plot taken by a multispectral camera in mid-June. Red and green colours indicate higher (*i.e.*, greater biomass and chlorophyll content) and lower (*i.e.*, lower biomass and chlorophyll content) NDVI values, respectively.

Table S1 Habitat, leaf duration, growth height, and rooting depth of the five study species according to Landolt et al. (2010) and Pignatti et al. (2017).

Species	Habitat	Leaf duration	Growth height	Rooting depth
Arctostaphylos uva-ursi	Montane and subalpine pine forests, rhododendron shrublands, screes	Evergreen	5-10 cm	50-100 cm
Vaccinium myrtillus	Forests (mainly dominated by Picea abies), heaths, shrublands, and subalpine pastures	Deciduous	15-50 cm	25-50 cm
Vaccinium vitis-idaea	Subalpine heaths; boreal forests	Evergreen	10-30 cm	5-15 cm
Calluna vulgaris	Heathlands, bogs	Evergreen	20-40 cm	25-50 cm
Salvia officinalis	Dry, rocky, Mediterranean slopes	Evergreen	30-70 cm	15-30 cm

Table S2 ANOVA results with the effects of species, substrate depth (plot), and their interaction on leaves, branches, and roots biomass.

Plant part	Factor	Df	Sum sq	Mean sq	F value	Pr(>F)
Leaves	Species	3	10562	3521	37.08	$1.34*10^{-13}$
	Plot	1	2168	2168	22.83	$1.21*10^{-5}$
	Species*Plot	2	4262	2131	22.45	$5.64*10^{-8}$
	Residuals	59	5602	95		
Branches	Species	4	2593.7	648.4	69.5	$< 2*10^{-16}$
	Plot	1	460.5	460.5	49.36	$4.95*10^{-10}$
	Species*Plot	4	2108.5	527.1	56.5	$< 2*10^{-16}$
	Residuals	85	793	9.3		
Roots	Species	4	13634	3408	55.32	$< 2*10^{-16}$
	Plot	1	3896	3896	63.23	$5.45*10^{-12}$
	Species*Plot	4	3490	872	14.16	$5.55*10^{-9}$
	Residuals	89	5483	62		

References:

Landolt, E. (2010). *Flora indicativa. Ökologische Zeigerwerte und biologische Kennzeichen zur Flora der Schweiz und der Alpen*, 2nd edition. Bern: Haupt Verlag.

Pignatti, S., Guarino, R., & La Rosa, M. (2017). *Flora d'italia* (Vol. 1).
